# Thicket Formation and Light Limitation Drive Patch‐Scale Vegetation Transitions in a Subtropical Savanna

**DOI:** 10.1002/ece3.73739

**Published:** 2026-06-02

**Authors:** Marina R. Khoza, Susanne Vetter

**Affiliations:** ^1^ Department of Botany Rhodes University Makhanda South Africa; ^2^ Copernicus Institute of Sustainable Development Utrecht University Utrecht the Netherlands

**Keywords:** bush encroachment, plant diversity, savanna ecosystems, savanna resilience, thicket expansion

## Abstract

Bush encroachment, in which C_3_ woody vegetation invades historically C_4_ grass‐dominated landscapes, is common in savannas worldwide. This process may lead to changes in the balance of tree and herbaceous species as well as transitions from open savannas to closed woodland or thicket states. When savannas are open, with high light availability, they support a high diversity of herbaceous species which maintain the overall function and resilience of the ecosystem. We analysed woody cover changes between 1949 and 2021 across 51 sites in a subtropical savanna in the Eastern Cape Province of South Africa (MAP ca. 700 mm). Most sites showed an increase in woody cover over the years. Low woody cover sites were significantly associated with *Vachellia karroo* trees, together with grass species such as *Sporobolus fimbriatus* , *Digitaria eriantha* , *Eragrostis chloromelas* and the forb *Hibiscus aethiopicus*. Densely wooded sites were characterised by woody species including *Carissa macrocarpa* , *Canthium ciliatum*, *Scolopia mundii*, *Dovyalis zeyheri* and *Afrocanthium mundianum*, alongside herbaceous species *Stipa dregeana* and *Berkheya onopordifolia*. The relationship between herbaceous basal cover and canopy conditions showed two data‐derived breakpoints, indicating that herbaceous basal cover changed at different rates along the tree‐density gradient. The first breakpoint was estimated at 19% woody cover (87% light transmittance; LAI 0.14), where basal cover began a sharp decline at the onset of encroachment and a second at 51% woody cover (63% light transmittance; LAI 0.88), where basal cover decreased steeply, coinciding with a compositional shift from *Vachellia karroo*‐dominated savanna toward evergreen thicket vegetation. Herbaceous species richness declined steeply in the densest canopies, indicating progressive loss of understory diversity as canopies closed. Together, these results suggest that maintaining woody cover below ca. 50% is crucial for preventing progression to a persistent closed‐canopy thicket state with a less flammable herbaceous layer.

## Graphical abstract

Bush encroachment drives a shift from savanna dominated by *Vachellia karroo* to thicket vegetation. As canopies close, light availability declines, herbaceous cover and species richness decrease, and shade‐tolerant species replace open‐savanna taxa. Woody cover above ca. 50% marks a critical transition toward a densely wooded closed thicket state.

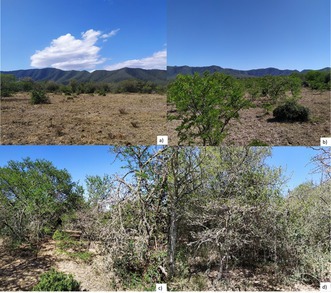

## 1 Introduction

Savannas are ancient ecosystems characterised by a continuous layer of graminoids (grasses and grass‐like plants) and a discontinuous layer of trees or shrubs (Bond and Parr 2010; House et al. 2003; Scholes and Archer 1997; Veldman 2016; Veldman et al. 2015). Trees in these environments are usually fire‐tolerant and range in density from scattered individuals to dense woodlands (House et al. 2003; Sankaran et al. 2005). In contrast to forests, plant diversity in savannas is greatest in the herbaceous layer, where light availability and disturbances from fire and herbivory maintain a diverse assemblage of grasses and forbs (Siebert and Dreber 2019; Van Coller et al. 2018; Wragg et al. 2018). The contribution of forb species to savanna diversity and ecosystem function has been overlooked, relative to trees and grasses, but their persistence in savannas is essential as they contribute not only to species diversity, but to savanna resilience. Due to their underground storage organs, many forb species can withstand dry conditions and disturbances such as grazing and fire, helping to sustain forage and post‐drought recovery when grass abundance is reduced (Siebert and Dreber 2019).

The balance between woody plants and the herbaceous layer can shift gradually or rapidly in response to changes in rainfall, herbivory, fire and rising atmospheric CO_2_ (Bond 2008; Buitenwerf et al. 2012; Kgope et al. 2010; Roques et al. 2001). A weakening of these controls on tree recruitment may lead to bush encroachment, which changes the growing conditions in the understory, with consequences for herbaceous species diversity, cover and overall ecosystem function (Wieczorkowski and Lehmann 2022). Woody‐herbaceous interactions range from competition for water, light and nutrients to facilitation through the amelioration of soil resources and microclimate (Belsky et al. 1989; Ludwig 2001; Scholes and Archer 1997). Trees and shrubs suppress heliophilc herbaceous species through shading, and also through root competition and by intercepting rainfall before it reaches the ground (Belsky et al. 1989; Dohn et al. 2013; Ludwig et al. 2004; Scholes and Archer 1997; Skhosana et al. 2023). At low density, large trees may facilitate grasses and forbs by improving soil fertility and moisture conditions under their canopies (Belsky et al. 1989, 1993; Dohn et al. 2013). However, over time, these ‘fertility islands’ can serve as nuclei for woody recruitment by concentrating seed rain and creating microhabitats that are sheltered from fire and water stress. This can lead to the initiation of woody clump formation and eventually canopy closure, which negatively affects herbaceous cover and diversity (Barnes and Archer 1996; Yarranton and Morrison 1974).

Light availability is a direct mechanism linking changes in canopy structure to herbaceous responses. As canopies become denser, leaf area index (LAI) increases and understory light transmittance declines, with predictable effects on herbaceous species. As shade increases, heliophilic herbaceous species decline and the herbaceous layer shifts in composition toward taxa that are shade‐tolerant (Hoffmann et al. 2012; Pilon et al. 2021). The dominant savanna grasses have the C_4_ photosynthetic pathway and are productive under high light (Wasilewska‐Dębowska et al. 2022). With increasing shade, an initial shift in C_4_ species composition, including shifts in photosynthetic subtype, is followed by a replacement with C_3_ grasses and very little grass cover of any kind in deep shade (Charles‐Dominique et al. 2018). Increasing tree cover thus results in a turnover in the herbaceous community from strongly light‐demanding species to an assemblage that is better adapted to shade and is less flammable.

Fire is a key ecological process in savannas, where it helps maintain the characteristic open tree‐canopy structure by restricting tree recruitment and supporting vegetation adapted to frequent burning and drought (Simon and Pennington 2012). Changes in herbaceous plant composition impact fire dynamics, because reductions in herbaceous biomass and shifts toward less flammable grass taxa can lower fire spread and intensity (Cardoso et al. 2018; Charles‐Dominique et al. 2018; Dormann et al. 2020). Two key ecological thresholds have been identified along the gradient from open savanna to closed‐canopy systems such as forests and thickets. The first threshold occurs when highly flammable C_4_ grasses begin to decline and are replaced by less flammable C_4_ grass species (Cardoso et al. 2018; Charles‐Dominique et al. 2018). The likelihood of a fire spreading is reduced but not entirely eliminated after this first shift. The second threshold represents the deep shade state, where woody cover becomes dense enough to exclude heliophilic species entirely. The system stabilises as a closed‐canopy forest or thicket after the second threshold, and this reduces the possibility of fire spreading further through the system (Charles‐Dominique et al. 2018; Lloyd and Veenendaal 2016).

Bush encroachment can take the form of increasing density of savanna trees (‘savanna thickening’) or involve the expansion of thicket or forest species into a savanna (‘thicket expansion’), resulting in a biome shift (Parr et al. 2014). In some savanna systems, thicket clumps form under large savanna trees. These expand and coalesce, with changing understory conditions favouring the recruitment of late‐successional, shade‐tolerant, broad‐leaved and evergreen species (Jamison‐Daniels et al. 2021; O'connor and Chamane 2012). In the Eastern Cape of South Africa, the encroaching tree *Vachellia karroo* is widely associated with savanna thickening, and it can also facilitate the establishment of thicket species, linking early encroachment stages to later closed‐canopy development (Acocks 1975; Dingaan and du Preez 2018; Nell et al. 2024; O'Connor 1995; O'Cconnor et al. 2014).

The structural and functional characteristics of savanna and forest have been well‐documented, and there has been a growing body of literature on the role of bush encroachment, particularly through clump formation, in transitions between these two states (Abreu et al. 2021; Jamison‐Daniels et al. 2021; Nell et al. 2024). Although there have been previous studies on changes in the light environment and herbaceous composition at the forest‐savanna ecotone (Cardoso et al. 2018; Charles‐Dominique et al. 2018), it remains poorly understood how increasing woody cover corresponds to coupled changes in (i) tree species composition, (ii) canopy structure and understory light availability (including LAI and light transmittance) and (iii) herbaceous species composition, cover and richness. This gap is important because the management of savanna systems relies on knowing when a site is still within a range where fire and grazing can maintain open structure versus when canopy‐driven feedbacks have led to a more persistent closed‐canopy state.

Here we examine how increasingly advanced stages of bush encroachment affect light availability and the composition, cover and species richness of the herbaceous layer in a subtropical savanna‐thicket mosaic (MAP: ca. 700 mm) in the Eastern Cape, South Africa. We used sequential aerial photographs to identify a gradient from open grassland to densely wooded patches representing different stages of bush encroachment across several decades. We hypothesised (i) that increasing woody canopy cover would be accompanied by a change in woody species composition from *Vachellia karroo*‐dominated savanna to more species‐rich evergreen thicket; (ii) that the more advanced stages of bush encroachment would be characterised by increasing LAI and decreased light transmittance; (iii) that decreased light transmittance would result in a compositional change in the herbaceous community from sun‐loving to shade‐tolerant grasses and forbs, with a decrease in C_4_ grass basal cover and reduced herbaceous species richness; and (iv) that these relationships would be characterised by non‐linear threshold behaviour, with abrupt shifts occurring once critical levels of woody cover, light transmittance, or LAI were reached. Crossing these thresholds would correspond to the decline of open savanna taxa and lead to the establishment of shade‐tolerant and inflammable thicket species, indicating a transition toward a closed‐canopy thicket state.

## 2 Materials and Methods

### 2.1 Study Area

**FIGURE 1:**
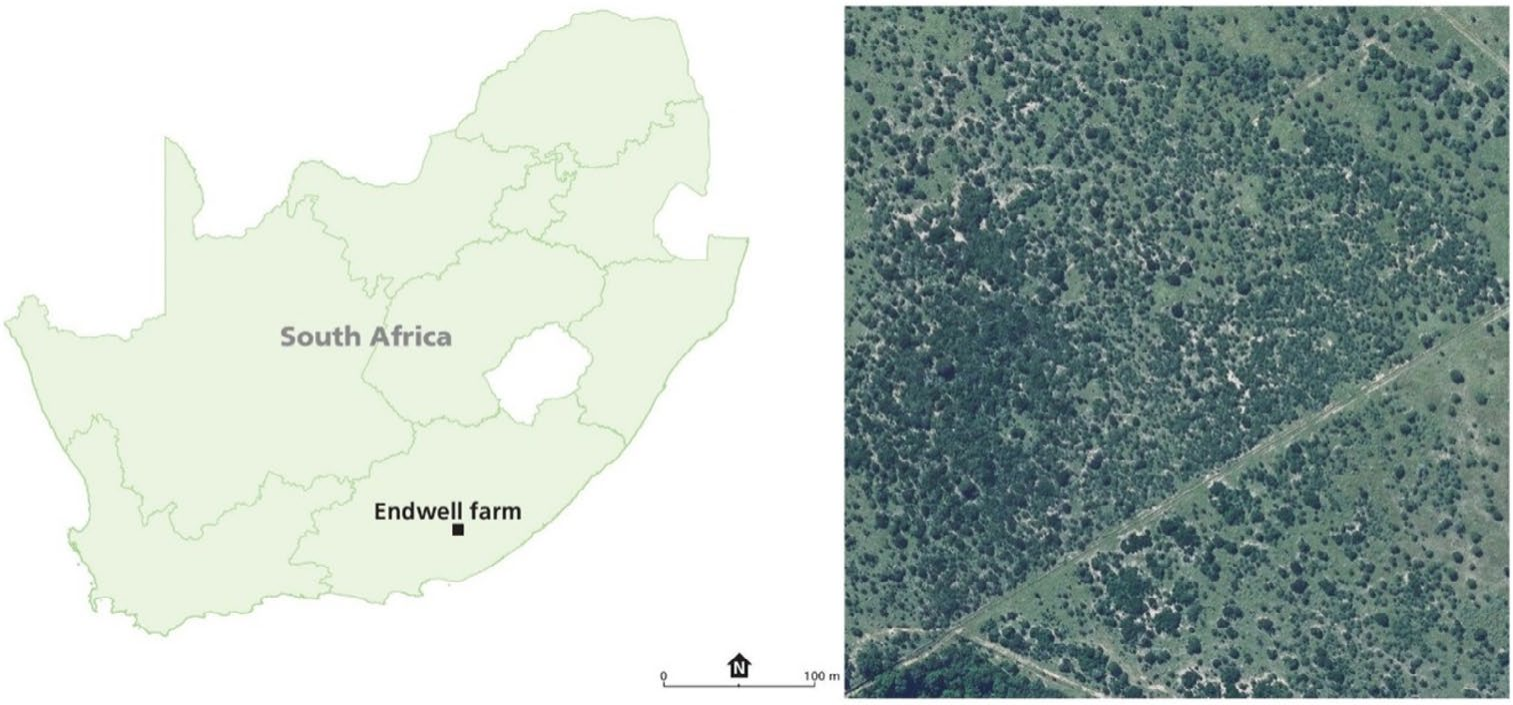
Location and aerial view of Endwell Farm, Eastern Cape, South Africa. The inset map (left) shows the position of Endwell Farm within South Africa. The aerial image (right) illustrates the study landscape, characterised by a heterogeneous mosaic of woody patches within savanna vegetation; scale bar = 100 m.

This study was conducted at Endwell Farm (ca. 850 ha) in the Eastern Cape Province of South Africa (Figure 1), which is managed for commercial cattle and wildlife production. The site is situated at an elevation of approximately 660 m above sea level and is located at 32°44′56.4″S, 26°27′41.6″E.

The climate at the study area is subtropical with warm, wet summers and cold, dry winters with occasional frost. The mean annual rainfall measured on the farm between 1924 and 2020 was ca. 700 mm with a coefficient of variation of 22%. Most rain is recorded between October and March and occurs in relatively few, but heavy rainfall events (Teague et al. 1981). Periods of rain are interrupted with intra‐season droughts, and during this time, topsoil moisture can drop below wilting point (Teague et al. 1981). The period of data collection was preceded by 4 years of below‐average rainfall in what farmers described as one of the worst droughts on record.

The predominant soil forms in the area are Glenrosa and Mispah derived from shales and sandstones of the Beaufort and Ecca series (Teague et al. 1981). While the soils are relatively eutrophic, they are usually shallow and stony and their high silt and fine sand content contributes to poor infiltration and drainage (Mills et al. 2009). The grasses in the area have high nutritional value that is retained through the dry winter season (Ellery et al. 1995). In most years, a high percentage of grass biomass is consumed by wild or domestic grazers, and fires are thus infrequent due to limited fuel loads.

**FIGURE 2:**
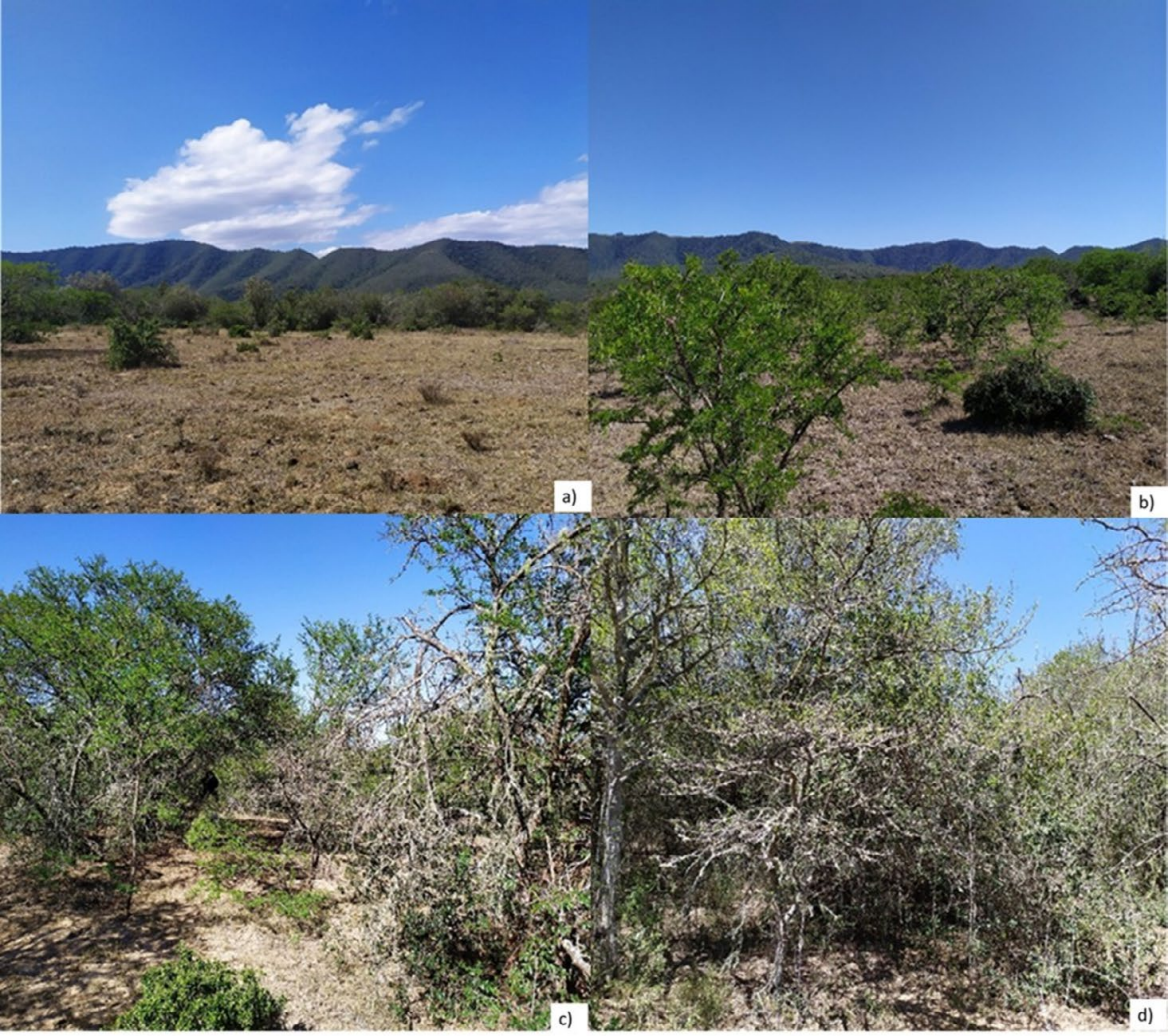
Photographs (taken in March 2020) of the study site show different woody cover states on Endwell Farm: (a) Open site (0%–15% woody cover) with minimal woody cover, representing a predominantly grassy savanna state (b) Low woody cover site (16%–30%) with scattered, immature *V. karroo* individuals. (c) Moderate woody cover site (31%–50%) showing increased density of *V. karroo* and a few thicket‐forming species. (d) High woody cover site (> 51%) representing a densely wooded state dominated by thicket‐forming species.

The vegetation at the sites consists of a mosaic of grassland, subtropical thicket, and savanna dominated by *V. karroo* (Mucina et al. 2006). Other common woody species include *Scutia myrtina*, *Ehretia rigida*, *Grewia occidentalis* , *Searsia longispina* and *Olea europaea* subsp. *africana*, which are characteristic of the Albany Thicket units (Kowie and Great Fish Thicket) described by Mucina et al. (2006). The grass layer is dominated by *Themeda triandra, Digitaria eriantha, Sporobolus fimbriatus* and *Cymbopogon species* (Mndela et al. 2023). Woody encroachment has been documented at the study site and takes the form of initial savanna densification followed by thicket clump formation and expansion, with *Vachellia karroo* typically acting as the founder species and thicket pioneers such as *Scutia myrtina* and *Gymnosporia buxifolia* initiating the formation of clumps (Nell et al. 2024; Figure 2).

### 2.2 Data Collection

#### 2.2.1 Woody Vegetation Cover Changes Over Seven Decades

The study sites were situated on relatively flat, low‐lying parts of the farm, where progressive woody encroachment has been evident in recent decades. To minimise environmental variation associated with topography, sites were selected along a common gentle slope. In total, 51 sites, each measuring 150 × 150 m, were identified from 2021 aerial photographs to represent a gradient of woody encroachment, ranging from open savanna to densely encroached thicket states. Woody cover within each 150 × 150 m site was required to be spatially uniform, ensuring that areas of low woody cover were not included within sites classified as high woody cover. The sites were also placed to avoid roads, drainage lines, and other man‐made or disturbed features to minimise effects of anthropogenic influences. The encroachment gradient was defined a priori using 2021 aerial imagery, and sites were selected in 2021 because this was the closest available representation of the vegetation at the time the study was conducted. We used stratified random sampling so that sites with varying woody cover were all included in the study. Densely wooded sites were rare and restricted to small, fragmented patches, which limited the number of sites with high woody cover.

Current woody cover was quantified for each of the 51 selected sites using a method adapted from Wigley et al. (2010) from 2021 aerial imagery. For each 150 × 150 m site, a regular grid was overlaid on the aerial imagery, producing 225 evenly spaced points across the site. Grid cells were therefore 10 × 10 m. At each point, the vegetation was classified as tree or grass, and the total number of ‘tree’ points determined the % woody cover.

To determine past changes in woody cover at these same 51 sites, aerial photographs of the farm in the years 1949, 1968, 1985, 2002 and 2013 were obtained from the National Geo‐Spatial Information (NGI) of South Africa. These images, which varied in scale and resolution, improved in quality in the more recent years (2002, 2013 and 2021) and were analysed at a scale that allowed clear distinction between woody and grassy vegetation components. The aerial imagery provided historical context for identifying long‐term changes in woody cover across the encroachment gradient and allowed us to test the hypothesis that areas with higher present‐day canopy cover have had a longer period of woody cover increase.

#### 2.2.2 Tree Cover and Composition

Vegetation composition at each of the 51 sites was assessed within a 15 × 6 m plot located at the centre of each 150 × 150 m site used to estimate woody cover. Each 15 m × 6 m sampling plot was divided into ten 3 m × 3 m subplots. We recorded tree species canopy cover and composition at each of the 51 sites during the summer growing season to ensure tree canopies were in full leaf, in November 2019, March 2020 and November 2020. Tree species were identified at the Schonland Herbarium (GRA) in Makhanda. We measured the height and canopy diameters (largest diameter and diameter perpendicular to the largest diameter) of all woody species with heights > 50 cm in each subplot of the 51 sites. The canopy area of each tree was estimated using the formula:

(1) $$A=\pi \frac{d_{1}}{2}\times \frac{d_{2}}{2}$$

where *d*_1_ and *d*_2_ are the maximum and perpendicular crown diameters (in meters), respectively. The total canopy area of all individuals of a given species was then summed and expressed as a proportion of the total plot area to obtain percent canopy cover per species.

#### 2.2.3 Understory Light Transmittance and Leaf Area Index Measurement

To determine the leaf area index (LAI) and the percentage of incident light reaching the understory at each of the 51 sites, we took hemispherical photographs. A Pentax K30 DSLR camera fitted with a fisheye converter lens (180° field of view) was used, with the lens facing upwards, producing circular photos. Hemispherical photographs were taken at four points spaced 3 m apart along the central (long) axis of each 15 × 6 m plot. These points corresponded to the corners of the 3 × 3 m subplots that fell along the plot's midline and were used to capture variation in light transmittance associated with the tree canopies located within the subplots. Each photograph was taken approximately 10 to 50 cm above the ground to ensure the grass layer did not affect the estimation of light transmittance. Hemispherical photographs were taken on overcast days, or when the sun was not directly overhead, to achieve a standardised sky background against which to compute canopy area.

We analysed photographs using Gap Light Analyser (GLA) 2.0 following the GLA manual (Frazer et al. 2000). The photos were configured by inputting the site location coordinates, growing season (September–April), sky region brightness and atmospheric conditions of the nearby town of Fort Beaufort. Leaf area index was derived from the angular distribution of canopy gap fraction (canopy openness) calculated by GLA.

#### 2.2.4 Sampling Herbaceous Composition and Cover

The composition of herbaceous species at each site was assessed within the same 15 × 6 m plots in which tree cover and composition, and light transmittance were measured. We sampled the cover of herbaceous species during the summer growing season from late February to March 2020. We identified flowering herbaceous species to the species level and estimated their percentage aerial cover in each 3 × 3 m subplot. The aerial cover of each species in each subplot was assigned a score of 1 (< 1%), 2 (1%–10%), or 3 (> 10%), so that the total abundance score for any species in a plot could range from 1 (< 1% cover in one subplot) to 30 (> 10% cover in all ten subplots).

Herbaceous basal cover was estimated using a pin‐drop frame—1 m long (Levy bridge; Levy and Madden 1933) fitted with 10 pins. The frame was placed at the center of each of the ten 3 m × 3 m subplots. For each pin that struck the rooted base of a plant, the species was recorded, while the remaining pins were recorded as having struck bare ground. Basal cover was calculated as follows:

(2) $$\%\text{basal cover}=\frac{\text{Number of pins that touch}\;a\;\text{plan}t^{'}s\;\text{base}}{\text{Total number of pins}}\times 100$$

### 2.3 Data Analyses

#### 2.3.1 Woody Cover Classification and Temporal Change

Changes in woody cover were assessed by categorising sites into four classes (open, low, moderate and high) using canopy cover estimates from historical and present aerial photographs. These categories were then related to visible differences in vegetation structure and the dominant functional types of woody plants, which we used to confirm thicket‐like sites (cf. Charles‐Dominique et al. 2015). We categorised woody cover states as follows: ‘open’ sites (0%–15% woody cover) corresponded to an open grassland state, ‘low’ woody cover sites (16%–30%) represented a savanna state dominated by scattered individual trees, ‘moderate’ woody cover sites (31%–0%) contained denser canopies, and ‘high’ woody cover sites (> 51%) represented closed‐canopy states (Figure 1). This high woody cover class agrees with the definition given by Puttick et al. (2014) for ‘thicket’ or ‘closed woodland’ in an area near our study site. Miettinen et al. (2015) similarly classified sites with 50% tree cover as ‘forests’ in their study of fire‐induced forest degradation in Brazil.

To visualise changes through time, we used a ridgeline plot in R, where each ridge represented the distribution of site‐level woody cover (or cover class values) for a given year. Ridgelines were generated using kernel density estimates to provide a smoothed representation of the distribution of woody cover across sites within each year.

#### 2.3.2 Cluster Analysis of Woody Species Composition and Indicator Species Analysis of Woody Community Clusters

Woody communities along the encroachment gradient were identified by hierarchical clustering of sites based on woody species composition. By defining woody community clusters first, we could then evaluate how variation in woody composition and canopy structure related to changes in the light environment and the herbaceous layer composition. Tree species cover across the entire plot was classified using the same cover categories as the herbaceous layer: 1 (< 1%), 2 (1%–10%) and 3 (> 10%). Categorical percentage scores for herbaceous species were assigned to each subplot and subsequently summed across subplots to derive a total value for the entire plot. A dendrogram was generated from the standardised tree species cover data using Ward's method (Ward Jr 1963) and the vegedist function from the vegan package in R (R Core Team 2023).

To test whether canopy structure and the associated light environment differed among woody community clusters, we analysed woody canopy cover (%), light transmittance (%) and leaf area index (LAI) as response variables using Kruskal–Wallis tests. To test which clusters differed from one another when overall effects were significant, we performed Dunn's pairwise post hoc comparisons with a Bonferroni correction.

Woody and herbaceous species that characterised the five woody community clusters were identified using the multipatt function in the indicspecies package (R), which implements Indicator Species Analysis (IndVal; Cáceres and Legendre 2009). This method estimates two conditional probabilities for each species. Component A, referred to as specificity, gives the probability that a sampled site belongs to the cluster given that the species was found at the site. Component B, referred to as the fidelity, shows the probability of finding the species at a site if it falls within a cluster. The indicator value is the product of the two components A and B and ranges from 0 to 1, and the ‘stat’ value is the square root of the indicator value, with higher values indicating stronger association with a cluster. Statistical significance was assessed using 1000 permutations of cluster labels to generate a null distribution of stat, and *p*‐values were calculated as the proportion of permutations in which stat ≥ the observed value. A species was considered an indicator when its maximum stat occurred for a given cluster and *p* ≤ 0.05.

To visualise the changes between woody community clusters and their indicator species in both the woody and herbaceous layers, we produced Sankey diagrams using the networkD3 package in R (R Core Team 2023). Only species that showed a significant association (*p* < 0.05) with one or more clusters in the Indicator Value (IndVal) analysis were included in the diagrams.

#### 2.3.3 Quantile Regression Analysis of Herbaceous Cover

To examine whether the response of herbaceous cover to increased woody cover and reduced light availability exhibited threshold behaviour, we fitted a linear quantile regression model with fixed breakpoints. The dependent variable was herbaceous basal cover, and the independent variables were woody cover (%), light transmittance (%) and LAI.

For each predictor, we specified two breakpoints, which partitioned the predictor range into three segments (low, intermediate, high). We defined the breakpoints (woody cover: 19.42% and 51.32%; transmittance: 62.76% and 86.56%; LAI: 0.14 and 0.88) from the empirical distribution of each predictor as the 25th and 75th percentiles of the observed values. We then fitted a linear model within each segment and found segment‐specific slopes to evaluate changes in direction and magnitude of the relationship across segments. Threshold‐like behaviour was inferred when slopes differed between adjacent segments, indicating a change in the basal cover‐predictor relationship around the specified breakpoint values.

#### 2.3.4 Herbaceous Species Richness Changes Along Woody Structural Gradients

To examine how herbaceous species richness varied along the woody structural gradients, we calculated site‐level richness as the number of herbaceous species recorded in each plot and related these metrics to woody cover, LAI and light transmittance. Relationships were visualised using scatterplots with smoothed fitted curves and 95% confidence intervals. Linear regression models were used as an initial analysis, followed by generalised additive models (GAMs) to account for potential non‐linear responses.

## 3 Results

### 3.1 Increasing Percentage Woody Cover Over Seven Decades

**FIGURE 3:**
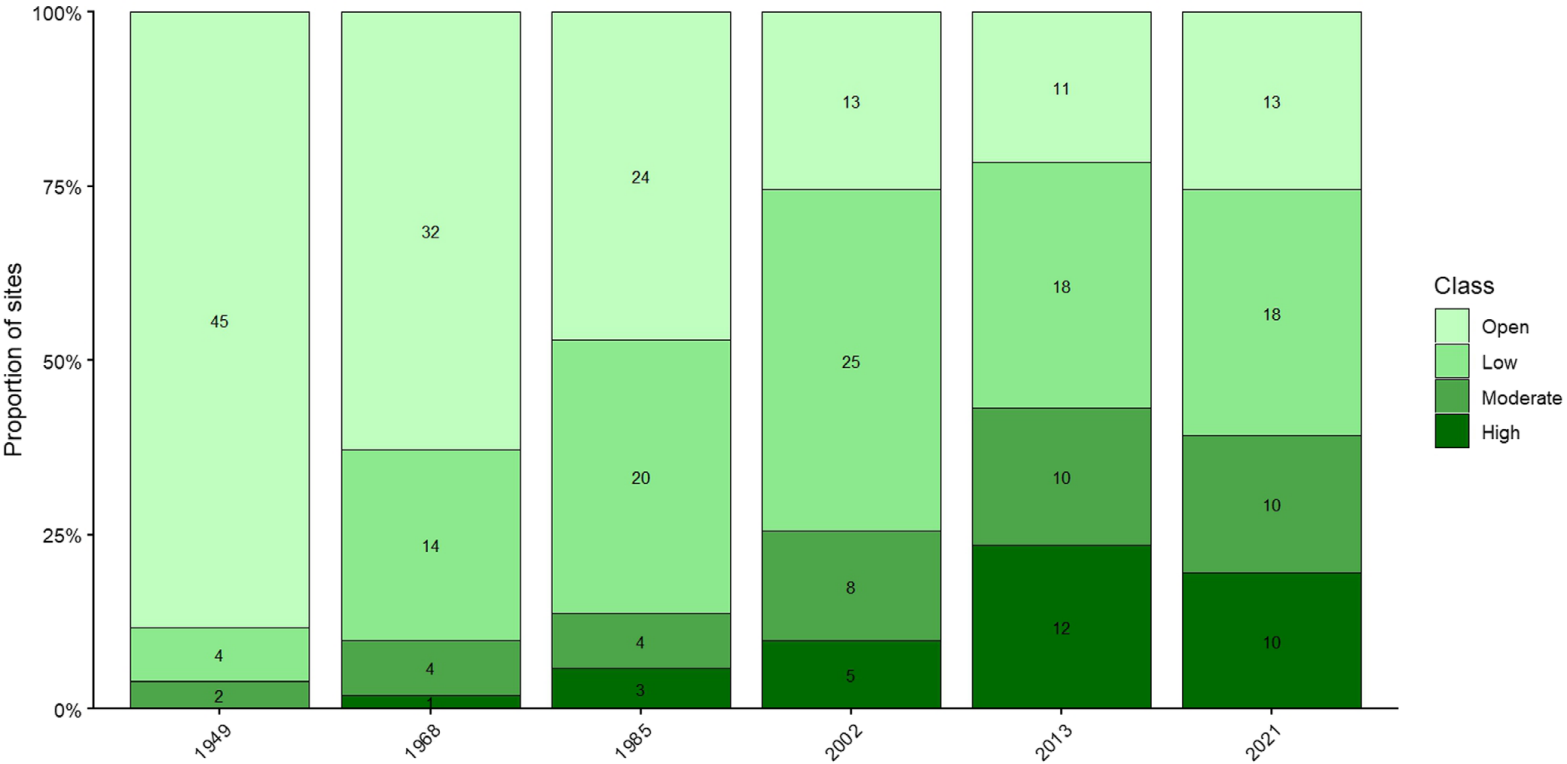
For each year shown (1949, 1968, 1985, 2002, 2013, 2021), stacked bars represent the proportion of sites assigned to one of four woody cover classes (Open, Low, Moderate, High; increasing woody cover from light to dark green). Values printed within each segment are the number of sites (counts) in that class for the corresponding year, and segments sum to the total number of sites assessed per year (51). The figure illustrates a progressive shift from predominantly open sites in earlier decades toward a greater proportion of moderate and high woody cover sites in later years.

**FIGURE 4:**
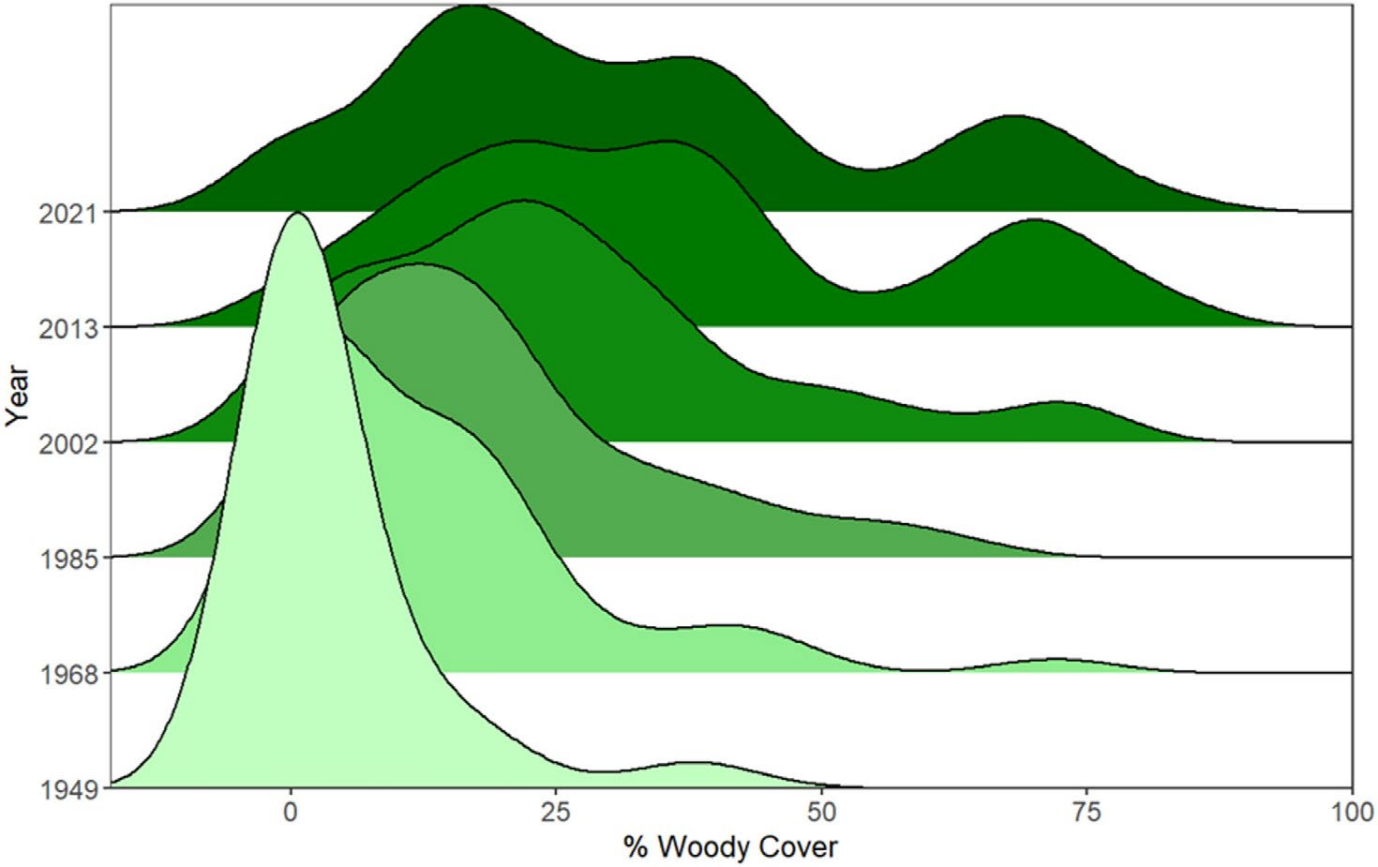
Density plots as ridges for aerial percent woody canopy cover estimated in 1949, 1968, 1985, 2002, 2013 and 2021.

Woody cover increased over time at most sites in this study (Figures 3 and 4). The number of open sites decreased from 45 in 1949 to 13 in 2021. No sites had high woody cover in 1949; by 2021, 10 sites were classified as high woody cover. The proportion of sites with moderate and high woody cover expanded notably after 2002. Early changes were modest: between 1949 and 1985, most shifts involved sites transitioning from open to the low woody cover class, and only occasionally into the moderate class (Figure 3). By 2002, most sites still had low woody cover, with the distribution peaking at approximately 25%, although a few sites had reached high cover. The subsequent decades, 2013 and 2021, showe a trimodal distribution of woody cover, with peaks at ca. 25%, 45% and 75%. Across all survey years, sites with 50%–60% cover remained uncommon (Figure 4).

### 3.2 Woody Composition and Its Effect on Light Transmittance and the Herbaceous Layer

**FIGURE 5:**
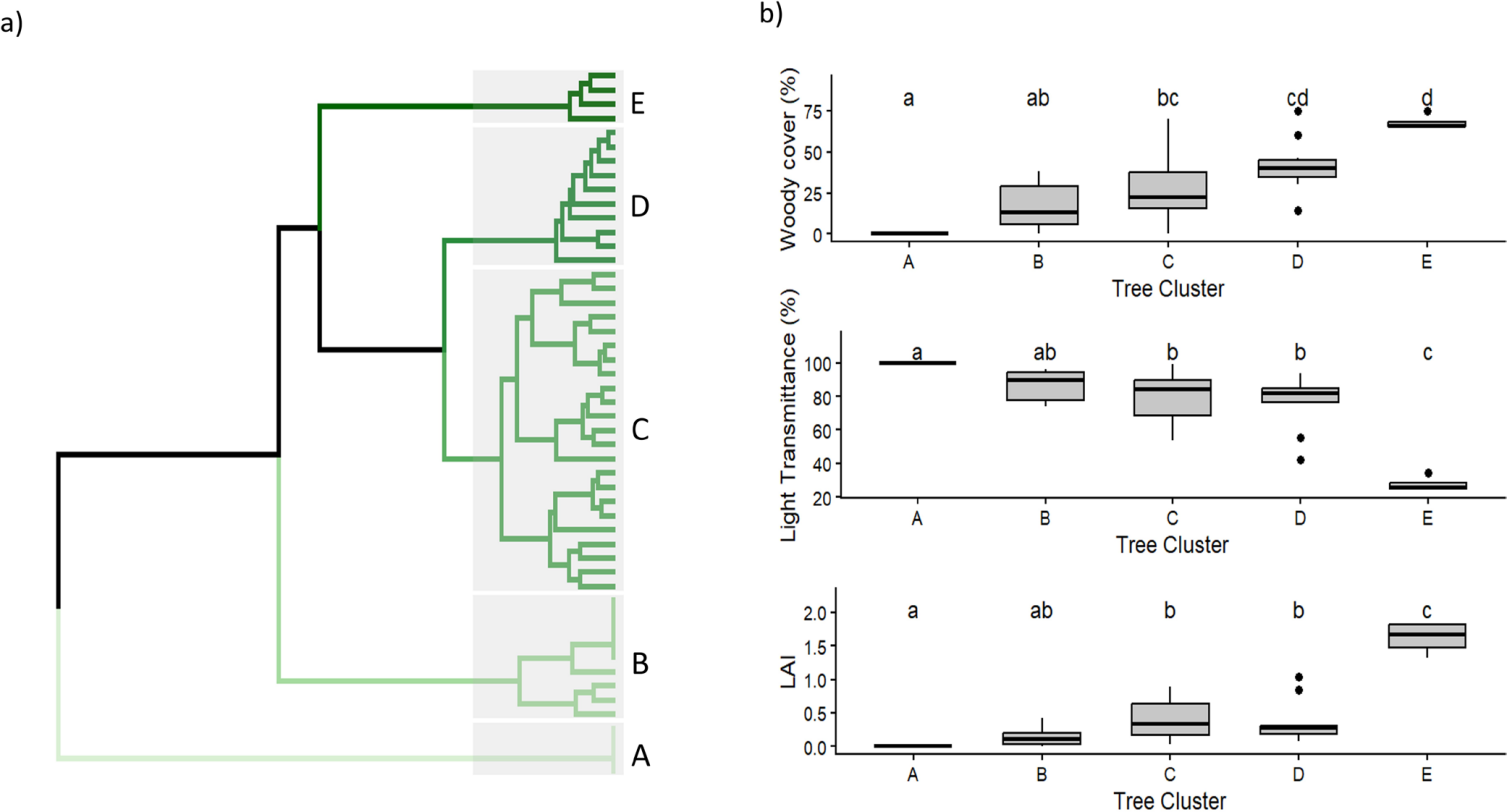
Woody community clusters and associated differences in canopy structure and light environment. (a) Hierarchical clustering dendrogram generated using Bray–Curtis distances and Ward's clustering method based on a matrix of tree species abundance scores. Five clusters are highlighted (Cluster A‐E) and represent a gradient in woody structure: A = grassland (no woody species recorded), B = open grassland/open savanna (low woody cover), C‐D = moderately wooded savannas and E = closed thicket (high woody cover). (b) Boxplots show the distribution of woody cover (%), light transmittance (%) and LAI for each cluster. Significant differences among clusters are indicated by different letters (a–c), where the same letter denotes no significant difference (*p* < 0.05).

Based on tree species composition, sites were classified into five distinct clusters (Figure 5a). Cluster A comprised sites with no recorded woody species, i.e., sites that were open grassland. Cluster B corresponded to open savanna communities with low woody cover (Figure 5b). Clusters C and D represent moderately wooded savannas with higher woody cover than clusters A and B. These clusters also differed significantly from the more open clusters in terms of light transmittance and LAI. Cluster E represented a closed thicket state with a distinct woody composition, significantly higher woody cover and LAI, and lower light transmittance.

**FIGURE 6:**
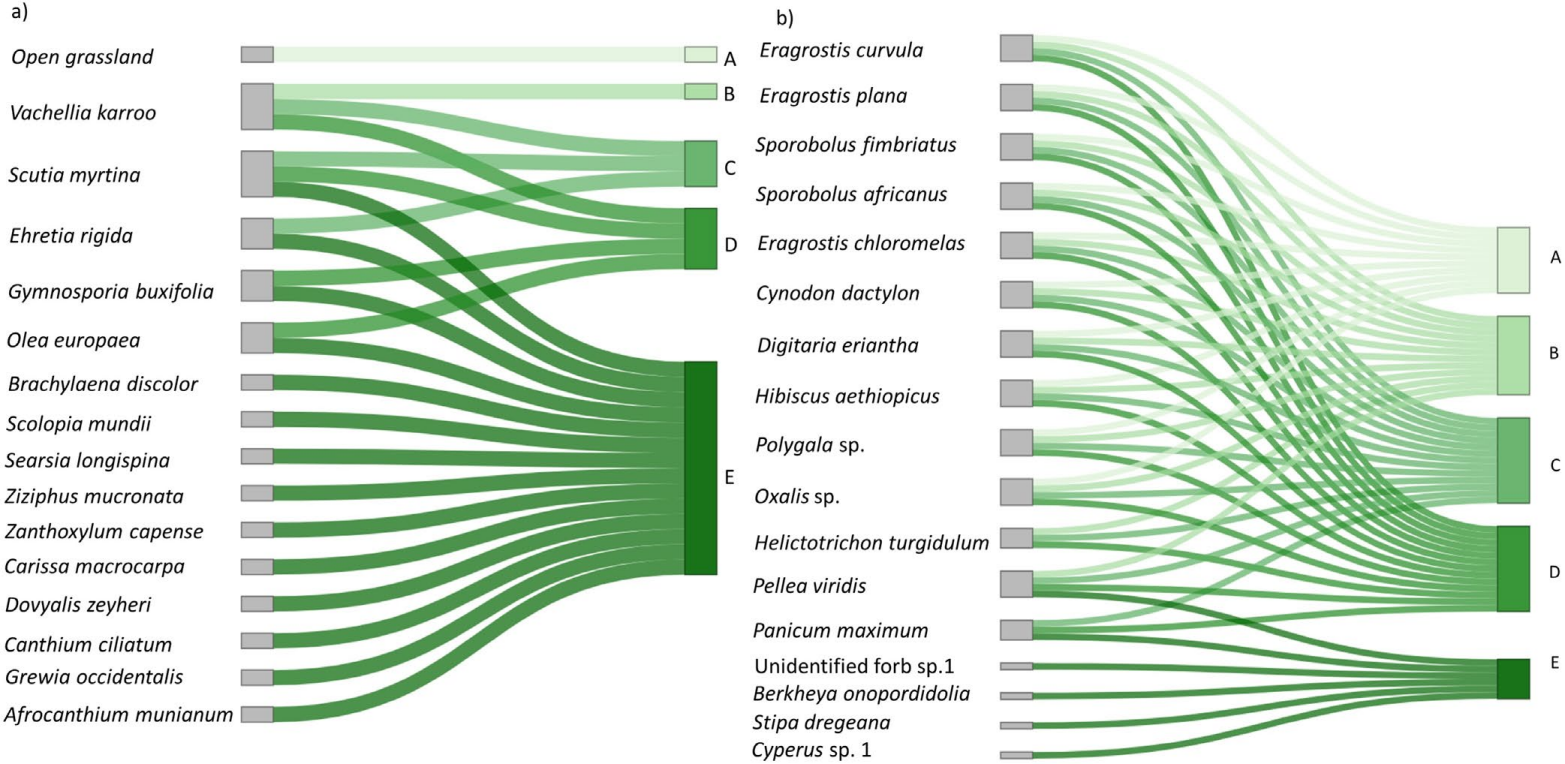
Sankey plots illustrating the contributions of (a) woody and (b) herbaceous indicator species to the five site clusters derived from the tree composition matrix. Nodes represent species and clusters, and connecting flows indicate significant species associations with each cluster. Flow colour follows the cluster gradient from (A to E), shown as a light to dark‐green sequence corresponding to increasing woody cover. Cluster (A) represents open sites with 0% woody cover, whereas clusters (B–E) represent progressively greater woody cover, from low (B) to high (E). *Vachellia karroo* was indicative of intermediate‐cover sites (clusters B–D), whereas several woody species, including *Afrocanthium mundianum*, *Canthium ciliatum* and *Dovyalis zeyheri*, were indicative of densely wooded sites in Cluster (E). Herbaceous species associated with more open conditions, particularly clusters (A–C), included *Eragrostis chloromelas* , *Hibiscus aethiopicus*, *Sporobolus fimbriatus* and *Digitaria eriantha* .

*Vachellia karroo* was the dominant tree species in this savanna system and was strongly associated with clusters B, C and D, which represented low to moderate woody cover (Table 1, Figure 6a). Within these intermediate clusters, *V. karroo* co‐occurred with species such as *Scutia myrtina*, while *Olea europaea* and *Gymnosporia buxifolia* were associated with the denser transitional clusters D and E. Cluster E was the most distinct in woody composition and characterised by species associated with dense thicket conditions, including *Carissa macrocarpa* , *Canthium ciliatum*, *Scolopia mundii*, *Grewia occidentalis* and *Dovyalis zeyheri*. Other characteristic species of this high woody cover cluster included *Afrocanthium mundianum*, *Searsia longispina*, *Ziziphus mucronata*, *Brachylaena discolour* and *Zanthoxylum capense*. *Vachellia karroo* was not characteristic of cluster E, marking the transition from open savanna to a more closed thicket state.

**TABLE 1:** Indicator species associated with vegetation clusters, showing indicator significance based on permutation tests where Component A (specificity or positive predictive) is sample estimate of the probability that the surveyed site belongs to the target group given that the species is present (Cáceres and Legendre 2009). Component B (fidelity or sensitivity) is sample estimate of the probability of finding the species in sites belonging to the site group. The stat value is the square root of the indicator value, which is the product of the two components ($\sqrt{A\times B}$) clusters. The open site group is indicated by the grey colour, and the light green to dark green colours indicate the groups B to E (low woody cover to high woody cover). Tree species names are in bold.

Species	Significance	A	B	C	D	E	Component A	Component B	Stat	Type
*Cynodon dactylon*	***						0.99	0.87	0.93	*Herb*
*Digitaria eriantha*	***						1.00	0.80	0.89	*Herb*
*Eragrostis chloromelas*	*						0.96	0.80	0.88	*Herb*
*Eragrostis curvula*	*						1.00	0.69	0.83	*Herb*
*Eragrostis plana*	**						1.00	0.80	0.89	*Herb*
*Hibiscus aethiopicus*	**						1.00	0.76	0.87	*Herb*
*Oxalis* sp.	**						1.00	0.78	0.88	*Herb*
*Polygala* sp.	**						1.00	0.76	0.87	*Herb*
*Sporobolus africanus*	*						0.99	0.78	0.88	*Herb*
*Sporobolus fimbriatus*	**						0.97	0.87	0.92	*Herb*
***Vachellia karroo***	***						0.98	1.00	0.99	**Tree**
*Helictotrichon turgidulum*	*						0.97	0.61	0.77	*Herb*
*Pellaea viridis*	*						1.00	0.70	0.83	*Herb*
***Ehretia rigida***	*						0.95	0.52	0.70	**Tree**
***Scutia myrtina***	***						1.00	0.95	0.97	**Tree**
*Panicum maximum*	*						0.92	0.65	0.77	*Herb*
***Gymnosporia buxifolia***	*						0.73	0.80	0.76	**Tree**
***Olea europaea***	***						0.90	0.93	0.92	**Tree**
***Afrocanthium mundianum***	**						1.00	0.60	0.78	**Tree**
***Brachylaena discolour***	*						0.85	0.40	0.58	**Tree**
***Canthium ciliatum***	***						0.94	0.80	0.87	**Tree**
***Canthium mundianum***	**						1.00	0.60	0.78	**Tree**
***Carissa macrocarpa***	***						1.00	0.80	0.89	**Tree**
***Dovyalis zeyheri***	***						0.83	0.80	0.82	**Tree**
***Grewia occidentalis***	***						0.90	0.80	0.85	**Tree**
***Scolopia mundii***	***						0.91	0.80	0.85	**Tree**
***Searsia longispina***	*						0.71	0.60	0.65	**Tree**
***Zanthoxylum capense***	*						0.80	0.40	0.57	**Tree**
***Ziziphus mucronata***	*						0.68	0.60	0.64	**Tree**
Unidentified forb sp. 1	*						0.88	0.40	0.59	*Herb*
*Berkheya onopordifolia*	*						0.86	0.40	0.59	*Herb*
*Cyperus* spp.	**						0.81	0.80	0.81	*Herb*
*Stipa dregeana*	**						0.77	0.60	0.68	*Herb*

*Note:* Significance levels: *p* < 0.05 (*), *p* < 0.01 (**), and *p* < 0.001 (*).

Herbaceous indicator species shifted along the woody cover gradient (Figure 6b and Table 1). In the open grassland cluster (A), no herbaceous species showed exclusive affinity for cluster A alone. Instead, several herbaceous species were associated with sites spanning open to moderately wooded conditions. These included the grasses *Cynodon dactylon* , *Eragrostis curvula* , *Sporobolus fimbriatus* , *Digitaria eriantha* , *Eragrostis plana* , *Eragrostis chloromelas* , *Sporobolus africanus* , and the forbs *Hibiscus aethiopicus*, *Polygala* sp., *Oxalis* sp., all of which were associated with clusters A–D. The grass *Helictotrichon turgidulum* was associated with clusters B–D. Only a few herbaceous species were significantly associated with the most wooded conditions. The grass *Panicum maximum* was associated with clusters C–E, and the fern *Pellaea viridis* was associated with clusters B–E, showing that they were associated with both shaded sites and sites with low woody cover. In cluster E, the species indicative of dense woody cover was an unidentified sedge species *Cyperus* sp. 1, the grass *Stipa dregeana*, and an unidentified forb sp. 1 (stat = 0.59, *p* < 0.05), and *Berkheya onopordifolia*.

### 3.3 Changes in Herbaceous Basal Cover and Biodiversity Along a Woody Cover, Light and LAI Gradient

**FIGURE 7:**
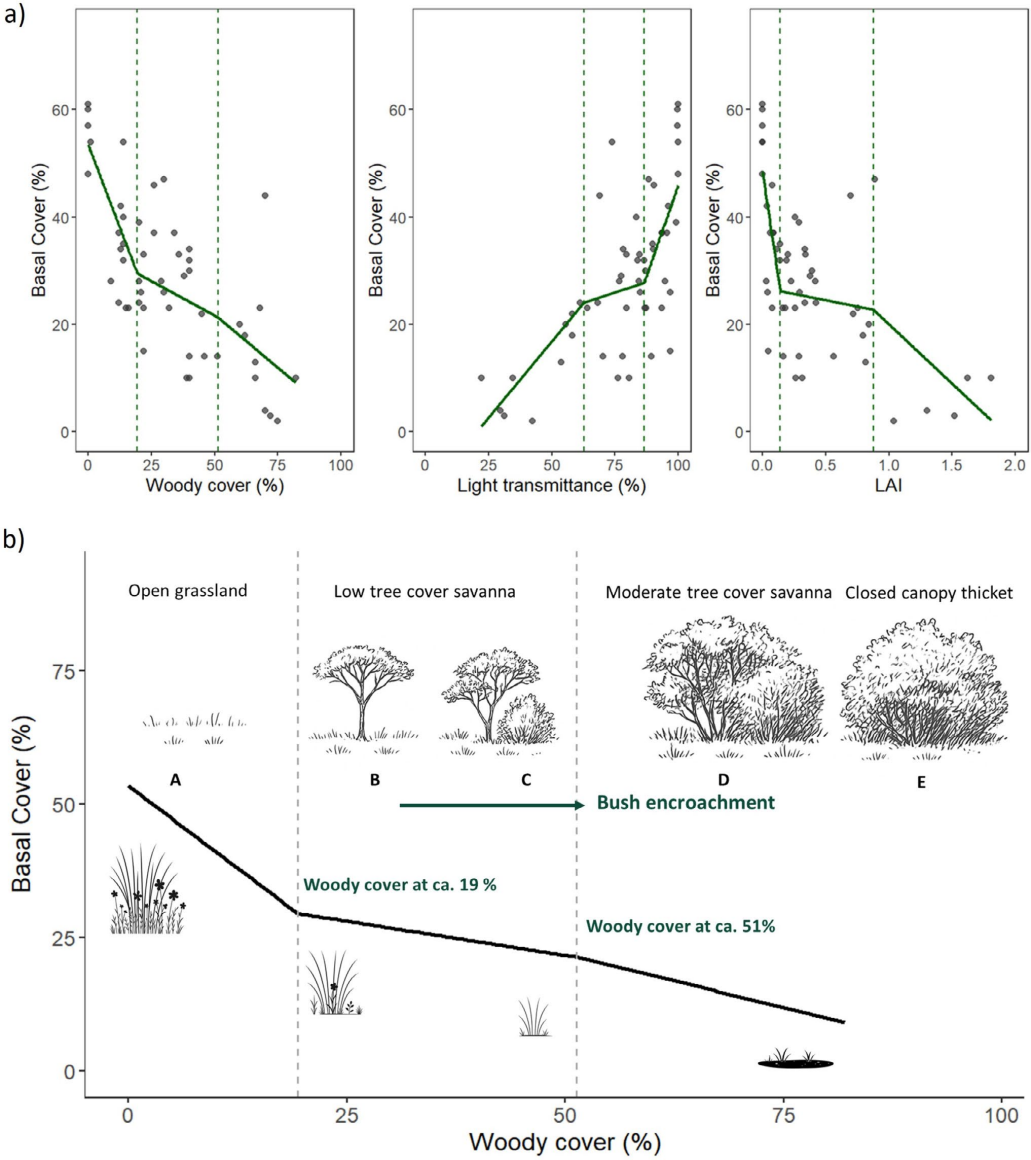
Relationship between herbaceous basal cover and canopy conditions along the bush encroachment gradient. (a) Herbaceous basal cover (%) in relation to woody cover (%), light transmittance (%) and leaf area index (LAI). Vertical dashed lines indicate 0.25 quantile thresholds of herbaceous basal cover at ca. 19% woody cover, ca. 87% light transmittance, and LAI of ca. 0.14 and the upper limits at the 0.75 quantile thresholds–51% woody cover, 63% light transmittance, and LAI of ca. 0.88. (b) The Schematic drawing follows the trends of increasing woody cover (%) and its impact on basal cover (%) of herbaceous species. Thresholds (dashed lines) indicate transitions between vegetation states: (A) open grassland, (B–C) low woody cover savanna, (D) moderate woody cover savanna and (E) closed‐canopy thicket. Upper and lower percentile thresholds highlight points where shifts in woody cover, light transmittance and LAI mark transitions associated with bush encroachment.

Herbaceous basal cover showed a non‐linear decline as percent woody canopy cover and LAI increased and as light transmittance increased (Figure 7a), reaching its lowest values in the densest thicket state (Figure 7a,b). Quantile regression revealed threshold conditions where herbaceous cover began to decline. At the lower quantile (τ = 0.25), basal cover declined rapidly up to 19% woody cover. This canopy cover corresponded to 87% light transmittance and LAI of 0.14. Above this threshold, herbaceous basal cover declined more gradually. At the upper quantile (τ = 0.75), a steeper decline in herbaceous basal cover was again observed above 51% woody cover, a light transmittance below 63%, and a LAI over 0.88. Increasing woody cover was therefore associated not only with distinct woody and herbaceous community composition (Figure 7a) but also a non‐linear decline in herbaceous cover as grassland and savanna transitioned to thicket (Figure 7b).

### 3.4 Declining Herbaceous Species Richness With Woody Densification

**FIGURE 8:**
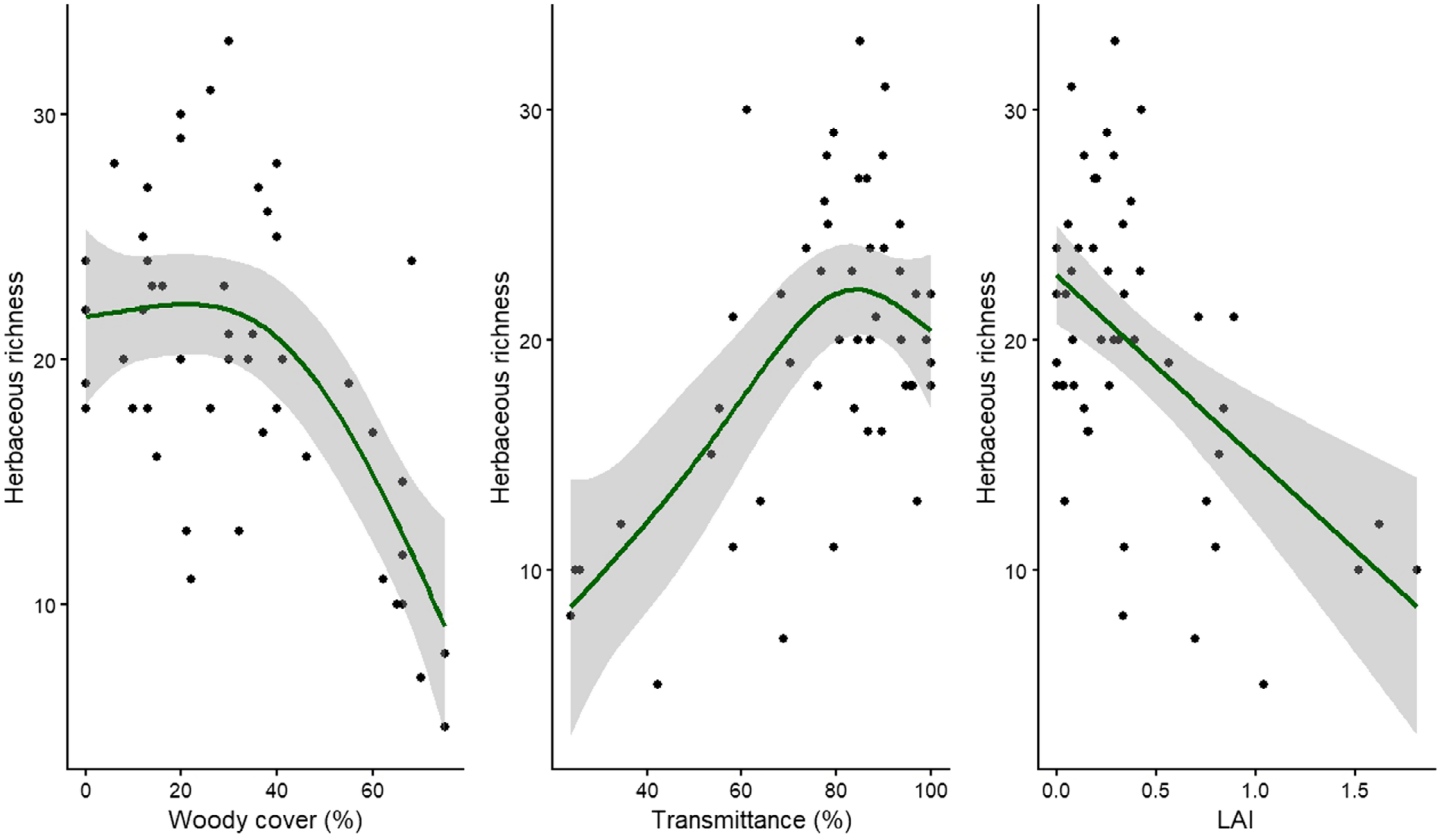
Relationships between herbaceous species richness (top row) and Shannon diversity (bottom row) and tree cover (%), light transmittance (%), and leaf area index (LAI). Points show site values; the solid line shows the fitted smooth with 95% confidence bands.

Herbaceous species richness was impacted by increasing woody cover (%) and LAI and a decline in light transmittance (Figure 8) similar to trends in percent herbaceous basal cover. Herbaceous species richness remained high under open to moderately wooded conditions, but declined sharply at higher woody cover. Richness remained high under open to moderately wooded conditions, but declined sharply at higher woody cover. Significant non‐linear relationships were detected between herbaceous species richness and woody cover (GAM: edf = 2.48, χ^2^ = 28.42, *p* = 3.02 × 10^−6^; adjusted *R* ^2^ = 0.36; deviance explained = 42%; *n* = 51), light transmittance (edf = 2.42, χ^2^ = 27.31, *p* = 1.16 × 10^−5^; adjusted *R* ^2^ = 0.33; deviance explained = 39.5%; *n* = 51) and LAI (edf = 1.87, χ^2^ = 16.18, *p* = 6.77 × 10^−4^; adjusted *R* ^2^ = 0.22; deviance explained = 26.5%; *n* = 51).

## 4 Discussion

4.1

Our data support the hypotheses that increasing woody cover represents a biome shift from open savanna to closed‐canopy thicket, where increasing woody cover is accompanied by compositional changes in woody species and a reduction in light availability. This in turn led to compositional changes in the herbaceous layer and a loss in herbaceous cover and richness characterised by threshold responses.

The pattern of increasing woody cover aligns with broader regional trends of bush encroachment documented in the Eastern Cape throughout the 20^th^ century and continuing into the early 21^st^ century (O'Cconnor et al. 2014). In 1949, most of the landscape was in an open grassland or savanna state, although some sites had moderate woody cover. The increase in woody cover appears to have accelerated between 1985 and 2002, and this could be linked to a range of factors, including the severe droughts of the early 1980s and the early 1990s (Masih et al. 2014). Droughts can influence encroachment by reducing grass biomass and continuity, which in turn lowers fuel loads and fire intensity in systems where fire constrains woody recruitment (Smit et al. 2024; Staver et al. 2019).

The distribution of woody cover across the decades in Figure 4 shows recurring modes rather than a continuous gradient. Across all decades, sites with approximately 50%–60% woody cover were rare, even though several sites transitioned from below to above this range. This pattern suggests that the 50%–60% woody cover range represents a transient state, corresponding with larger scale analyses showing discontinuities in tree cover distributions linked to fire‐vegetation feedbacks (Hirota et al. 2011; Staver et al. 2011). In our dataset, rarity of sites in this range suggests that this interval is a relatively brief transition between more persistent open and closed‐canopy states.

As sites transitioned from open savanna to increasingly dense thicket, LAI increased and light transmittance decreased. In addition to increasing tree‐density, changes in the functional traits of the dominant woody species affect ground‐layer conditions by influencing the availability of light and space (Charles‐Dominique et al. 2015, 2018). Savanna species such as *V. karroo* are usually deciduous and have sparse canopies with a spreading canopy structure and bipinnate compound leaves that allow light penetration to the understory (Gignoux et al. 2016; Ratnam et al. 2011). A richer herbaceous layer, including C_4_ grasses that are adapted to higher light levels, is supported by the varied light environment this growth type produces in savannas (Charles‐Dominique et al. 2018).

The transition from savanna to thicket and the accompanying decrease in light availability were accompanied by shifts in the herbaceous community. Savanna sites dominated by *V. karroo* were associated with a relatively diverse herbaceous assemblage, including C_4_ grass species such as *Digitaria eriantha* , *Sporobolus fimbriatus* , *Sporobolus africanus* and *Eragrostis plana* , and the forbs *Oxalis* sp. and *Hibiscus aethiopicus*, indicating that increases in woody cover did not trigger an immediate reorganisation of the herbaceous layer. Many of these species persisted across the lower to intermediate woody cover clusters, suggesting a gradual rather than abrupt response to encroachment. Substantial compositional changes were only evident in the denser woody classes dominated by woody thicket species, where species such as the C_3_ grass *Stipa dregeana* and sedge taxa became more characteristic. The moderately shade‐tolerant C_4_ grass *Panicum maximum* was associated with several woody cover clusters, consistent with its ability to persist under a broader range of light conditions.

Few grass species in savannas thrive in shaded environments (Belsky et al. 1993; Pilon et al. 2021; Solofondranohatra et al. 2021). Overall, a successional sequence with a clear pattern of turnover from savanna communities to thicket‐dominated states can be deduced from the changes in the composition of woody and herbaceous species as woody cover increases (as documented by Jamison‐Daniels et al. 2021; Nell et al. 2024).

Under conditions of high rainfall and low grazing pressure, fire can maintain the open savanna state, but this becomes less likely as woody cover increases and herbaceous biomass declines. An initial steep decline in herbaceous basal cover occurred as woody cover increased from 0% to 19%, corresponding to savanna thickening predominantly by *V. karroo*. This phase primarily reduced herbaceous species basal cover, with limited change in both tree and herbaceous species composition. This early loss of herbaceous cover may have reduced fine‐fuelled loads and continuity, weakening the capacity of fire to limit further woody expansion.

Between approximately 19% and 51% woody cover, herbaceous basal cover declined more gradually with increasing woody cover, while woody composition started the shift toward thicket formation. The grass layer in this state is still dominated by flammable grasses that can carry a fire if sufficient rain and low grazing allow enough biomass to be accumulated. Above approximately 51% woody cover, sites were increasingly charecterised by thicket and dry forest species, accompanied by higher LAI (> 0.88) and light transmittance (< 63%). Beyond this threshold, herbaceous basal cover dropped more sharply, and grass composition changed rapidly with heliophilic C_4_ grasses being replaced by the more shade‐tolerant *P. maximum* and the C_3_ grass *Stipa dregeana*. In the absence of fire or heavy browsing, this state is likely to progress quite rapidly toward the denser thicket states that are difficult to reverse (Parr et al. 2012) as woody plants grow bigger and recruit.

The two thresholds marked by steep declines in herbaceous basal cover, correspond closely with those reported in other savanna systems (Cardoso et al. 2018; Charles‐Dominique et al. 2015; Hoffmann et al. 2012). Charles‐Dominique et al. (2018) identified two key transitions in Hluhluwe‐iMfolozi Park: a ‘fire suppression threshold’ at a LAI of ca. 0.5, indicating reduced grass flammability and decreased fire spread, and a second threshold representing the ‘deep shade threshold’ at an LAI of ca. 1.5, associated with a shift from C_4_ to C_3_ grass dominance. Hoffmann et al. (2012) and Cardoso et al. (2018) also described sequential thresholds in mesic tropical savannas and Cerrado ecotones, where an initial decline in grass flammability and cover precedes an abrupt transition to a forested state. At our study site, the upper threshold observed at LAI ca. 0.88 falls below the deep shade threshold reported for mesic savannas, likely reflecting the higher light demand of C_4_ grasses, and the earlier onset of thicket formation under subtropical conditions at this site.

Herbaceous species richness decreased steadily under low to moderate woody cover but declined steeply once woody cover exceeded approximately 40%, coinciding with increasing LAI and reduced understory light. This pattern aligns with studies showing that increasing woody encroachment reduces herbaceous species richness and re‐organises understory communities (Ratajczak et al. 2012). The non‐linear relationships suggest that an increase in woody cover and a reduction in light is not met by an immediate decline in herbaceous species richness as both shade‐tolerant and heliophilic herbaceous species co‐occur. Peterson and Reich (2008) found that savannas with intermediate and spatially variable canopy cover supported higher understory species richness because patchy shading created a wider range of habitats for plants. This could explain the high species richness that is still present as woody cover increases moderately in our study.

## 5 Conclusion

In our study, sites have shifted from open savanna to states with moderate woody cover and, in some cases, to dense thicket vegetation characterised by distinct, thicket‐affiliated woody assemblages. Where canopy closure has developed, light availability is reduced and herbaceous basal cover together with herbaceous species richness is reduced. In these later stages of encroachment, the savanna tree, *V. karroo* was either sparse or absent, with sites becoming more dominated by thicket species such as *C. macrocarpa, D. zeyheri, S. mundii, A. mundianum and C* *. ciliatum* . Herbaceous basal cover declined steadily at moderate levels of less than 51% woody cover, supporting the idea that low to moderate tree densities maintain a reduced grass layer that can still fuel fires. The decrease of herbaceous species beyond 51%, however, indicates a change toward a more sheltered environment for herbaceous species, less flammable thicket state that may be difficult to reverse. From a management perspective, maintaining a woody cover below ca. 50% that allows > ca. 60% transmittance of sunlight to the understory is crucial for preventing progression to a closed‐canopy thicket state. To ensure a thriving herbaceous layer, woody cover should be maintained below approximately 20%, with pioneer species controlled through fire or browsing. Early application of management techniques such as prescribed burning and targeted browsing is necessary to avoid the development and expansion of thicket clumps, which are more expensive to manage and drive the system closer to the point at which fire loses its effectiveness. To guide long‐term conservation and adaptive management in subtropical regions, future studies should measure the savanna systems' resilience and capacity for recovery at various stages of woody encroachment.

## Author Contributions

**Marina R. Khoza:** conceptualization (equal), data curation (equal), formal analysis (equal), funding acquisition (equal), investigation (equal), methodology (equal), project administration (equal), resources (equal), software (equal), supervision (equal), validation (equal), visualization (equal), writing – original draft (equal), writing – review and editing (equal). **Susanne Vetter:** conceptualization (equal), data curation (equal), formal analysis (equal), funding acquisition (equal), investigation (equal), methodology (equal), project administration (equal), resources (equal), software (equal), supervision (equal), validation (equal), visualization (equal), writing – original draft (equal), writing – review and editing (equal).

## Conflicts of Interest

The authors declare no conflicts of interest.

## Data Availability

Data supporting the results of this study are available in: https://doi.org/10.5281/zenodo.1951193.

## References

[ece373739-bib-0001] Abreu, R. C. , G. Durigan , A. C. Melo , N. A. Pilon , and W. A. Hoffmann . 2021. “Facilitation by Isolated Trees Triggers Woody Encroachment and a Biome Shift at the Savanna–Forest Transition.” Journal of Applied Ecology 58, no. 11: 2650–2660.

[ece373739-bib-0002] Acocks, J. P. H. 1975. “Veld Types of South Africa.”

[ece373739-bib-0003] Barnes, P. W. , and S. Archer . 1996. “Influence of an Overstorey Tree ( *Prosopis glandulosa* ) on Associated Shrubs in a Savanna Parkland: Implications for Patch Dynamics.” Oecologia 105, no. 4: 493–500. 28307142 10.1007/BF00330012

[ece373739-bib-0004] Belsky, A. , R. Amundson , J. Duxbury , S. Riha , A. Ali , and S. Mwonga . 1989. “The Effects of Trees on Their Physical, Chemical and Biological Environments in a Semi‐Arid Savanna in Kenya.” Journal of Applied Ecology 26: 1005–1024.

[ece373739-bib-0005] Belsky, A. , S. Mwonga , and J. Duxbury . 1993. “Effects of Widely Spaced Trees and Livestock Grazing on Understory Environments in Tropical Savannas.” Agroforestry Systems 24: 1–20.

[ece373739-bib-0006] Bond, W. J. 2008. “What Limits Trees in C4 Grasslands and Savannas?” Annual Review of Ecology, Evolution, and Systematics 39, no. 1: 641–659.

[ece373739-bib-0007] Bond, W. J. , and C. L. Parr . 2010. “Beyond the Forest Edge: Ecology, Diversity and Conservation of the Grassy Biomes.” Biological Conservation 143, no. 10: 2395–2404.

[ece373739-bib-0008] Buitenwerf, R. , W. Bond , N. Stevens , and W. Trollope . 2012. “Increased Tree Densities in South African Savannas: > 50 Years of Data Suggests CO_2_ as a Driver.” Global Change Biology 18, no. 2: 675–684.

[ece373739-bib-0009] Cáceres, M. D. , and P. Legendre . 2009. “Associations Between Species and Groups of Sites: Indices and Statistical Inference.” Ecology 90, no. 12: 3566–3574. 20120823 10.1890/08-1823.1

[ece373739-bib-0010] Cardoso, A. W. , I. Oliveras , K. A. Abernethy , et al. 2018. “Grass Species Flammability, Not Biomass, Drives Changes in Fire Behavior at Tropical Forest‐Savanna Transitions.” Frontiers in Forests and Global Change 1: 6.

[ece373739-bib-0011] Charles‐Dominique, T. , G. F. Midgley , K. W. Tomlinson , and W. J. Bond . 2018. “Steal the Light: Shade vs Fire Adapted Vegetation in Forest–Savanna Mosaics.” New Phytologist 218, no. 4: 1419–1429. 29604213 10.1111/nph.15117

[ece373739-bib-0012] Charles‐Dominique, T. , A. Staver , G. Midgley , and W. Bond . 2015. “Functional Differentiation of Biomes in an African Savanna/Forest Mosaic.” South African Journal of Botany 101: 82–90.

[ece373739-bib-0013] Dingaan, M. , and P. J. du Preez . 2018. “Vachellia (Acacia) Karroo Communities in South Africa: An Overview.” Pure and Applied Biogeography 20: 109–141.

[ece373739-bib-0014] Dohn, J. , F. Dembélé , M. Karembé , A. Moustakas , K. A. Amévor , and N. P. Hanan . 2013. “Tree Effects on Grass Growth in Savannas: Competition, Facilitation and the Stress‐Gradient Hypothesis.” Journal of Ecology 101, no. 1: 202–209.

[ece373739-bib-0015] Dormann, C. F. , M. Bagnara , S. Boch , et al. 2020. “Plant Species Richness Increases With Light Availability, but Not Variability, in Temperate Forests Understorey.” BMC Ecology 20: 1–9. 32727542 10.1186/s12898-020-00311-9 PMC7392730

[ece373739-bib-0064] Ellery, W. , R. Scholes , and M. Scholes . 1995. “The Distribution of Sweetveld and Sourveld in South Africa's Grassland Biome in Relation to Environmental Factors.” African Journal of Range & Forage Science 12, no. 1: 38–45.

[ece373739-bib-0016] Frazer, G. W. , J. Trofymow , and K. P. Lertzman . 2000. “Canopy Openness and Leaf Area in Chronosequences of Coastal Temperate Rainforests.” Canadian Journal of Forest Research 30, no. 2: 239–256.

[ece373739-bib-0017] Gignoux, J. , S. Konaté , G. Lahoreau , X. Le Roux , and G. Simioni . 2016. “Allocation Strategies of Savanna and Forest Tree Seedlings in Response to Fire and Shading: Outcomes of a Field Experiment.” Scientific Reports 6, no. 1: 38838. 28000732 10.1038/srep38838 PMC5175285

[ece373739-bib-0018] Hirota, M. , M. Holmgren , E. H. Van Nes , and M. Scheffer . 2011. “Global Resilience of Tropical Forest and Savanna to Critical Transitions.” Science 334, no. 6053: 232–235. 21998390 10.1126/science.1210657

[ece373739-bib-0019] Hoffmann, W. A. , E. L. Geiger , S. G. Gotsch , et al. 2012. “Ecological Thresholds at the Savanna‐Forest Boundary: How Plant Traits, Resources and Fire Govern the Distribution of Tropical Biomes.” Ecology Letters 15, no. 7: 759–768. 22554474 10.1111/j.1461-0248.2012.01789.x

[ece373739-bib-0020] House, J. I. , S. Archer , D. D. Breshears , R. J. Scholes , and NCEAS Tree–Grass Interactions Participants . 2003. “Conundrums in Mixed Woody–Herbaceous Plant Systems.” Journal of Biogeography 30, no. 11: 1763–1777.

[ece373739-bib-0021] Jamison‐Daniels, S.‐L. , W. D. Kissling , M. Botha , M. A. Harris , C. E. Gordon , and M. Greve . 2021. “The Role of Deterministic Succession During Forest Development Within a Southern African Savanna.” Biotropica 53, no. 2: 466–476.

[ece373739-bib-0022] Kgope, B. S. , W. J. Bond , and G. F. Midgley . 2010. “Growth Responses of African Savanna Trees Implicate Atmospheric [CO_2_] as a Driver of Past and Current Changes in Savanna Tree Cover.” Austral Ecology 35, no. 4: 451–463.

[ece373739-bib-0023] Levy, E. B. , and E. Madden . 1933. “The Point Method of Pasture Analysis.”

[ece373739-bib-0024] Lloyd, J. , and E. Veenendaal . 2016. “Are Fire Mediated Feedbacks Burning Out of Control?” Biogeosciences Discussions 2016: 1–20.

[ece373739-bib-0025] Ludwig, F. 2001. Tree‐Grass Interactions on an East African Savanna: The Effects of Facilitation, Competition, and Hydraulic Lift. Wageningen University and Research.

[ece373739-bib-0026] Ludwig, F. , T. Dawson , H. Prins , F. Berendse , and H. De Kroon . 2004. “Below‐Ground Competition Between Trees and Grasses May Overwhelm the Facilitative Effects of Hydraulic Lift.” Ecology Letters 7, no. 8: 623–631.

[ece373739-bib-0027] Masih, I. , S. Maskey , F. Mussá , and P. Trambauer . 2014. “A Review of Droughts on the African Continent: A Geospatial and Long‐Term Perspective.” Hydrology and Earth System Sciences 18, no. 9: 3635–3649.

[ece373739-bib-0028] Miettinen, J. , Y. E. Shimabukuro , R. Beuchle , et al. 2015. “On the Extent of Fire‐Induced Forest Degradation in Mato Grosso, Brazilian Amazon, in 2000, 2005 and 2010.” International Journal of Wildland Fire 25, no. 2: 129–136.

[ece373739-bib-0029] Mills, A. , M. Fey , J. Donaldson , S. Todd , and L. Theron . 2009. “Soil Infiltrability as a Driver of Plant Cover and Species Richness in the Semi‐Arid Karoo, South Africa.” Plant and Soil 320, no. 1: 321–332.

[ece373739-bib-0030] Mndela, M. , S. Moss , B. Gusha , et al. 2023. “Functional Trait Responses of C4 Bunchgrasses to Fire Return Intervals in the Semi‐Arid Savanna of South Africa.” Diversity 15, no. 12: 1201.

[ece373739-bib-0031] Mucina, L. , M. C. Rutherford , L. W. Powrie , et al. 2006. Vegetation Atlas of South Africa, Lesotho and Swaziland, 748–789. South African National Biodiversity Institute.

[ece373739-bib-0032] Nell, R. , T. Pillay , and S. Vetter . 2024. “Reconstructing Thicket Clump Formation Using Association Rules Analysis.” Journal of Vegetation Science 35, no. 3: e13265.

[ece373739-bib-0034] O'Cconnor, T. G. , J. R. Puttick , and M. T. Hoffman . 2014. “Bush Encroachment in Southern Africa: Changes and Causes.” African Journal of Range & Forage Science 31, no. 2: 67–88.

[ece373739-bib-0035] O'Connor, T. 1995. “Transformation of a Savanna Grassland by Drought and Grazing.” African Journal of Range & Forage Science 12, no. 2: 53–60.

[ece373739-bib-0033] O'connor, T. , and S. Chamane . 2012. “Bush Clump Succession in Grassland in the Kei Road Region of the Eastern Cape, South Africa.” African Journal of Range & Forage Science 29, no. 3: 133–146.

[ece373739-bib-0036] Parr, C. L. , E. F. Gray , and W. J. Bond . 2012. “Cascading Biodiversity and Functional Consequences of a Global Change–Induced Biome Switch.” Diversity and Distributions 18, no. 5: 493–503.

[ece373739-bib-0037] Parr, C. L. , C. E. Lehmann , W. J. Bond , W. A. Hoffmann , and A. N. Andersen . 2014. “Tropical Grassy Biomes: Misunderstood, Neglected, and Under Threat.” Trends in Ecology & Evolution 29, no. 4: 205–213. 24629721 10.1016/j.tree.2014.02.004

[ece373739-bib-0038] Peterson, D. W. , and P. B. Reich . 2008. “Fire Frequency and Tree Canopy Structure Influence Plant Species Diversity in a Forest‐Grassland Ecotone.” Plant Ecology 194, no. 1: 5–16.

[ece373739-bib-0039] Pilon, N. A. , G. Durigan , J. Rickenback , et al. 2021. “Shade Alters Savanna Grass Layer Structure and Function Along a Gradient of Canopy Cover.” Journal of Vegetation Science 32, no. 1: e12959.

[ece373739-bib-0040] Puttick, J. R. , M. T. Hoffman , and J. Gambiza . 2014. “The Influence of South Africa's Post‐Apartheid Land Reform Policies on Bush Encroachment and Range Condition: A Case Study of Fort Beaufort's Municipal Commonage.” African Journal of Range & Forage Science 31, no. 2: 135–145.

[ece373739-bib-0041] R Core Team . 2023. R: A Language and Environment for Statistical Computing. R Foundation for Statistical Computing.

[ece373739-bib-0042] Ratajczak, Z. , J. B. Nippert , and S. L. Collins . 2012. “Woody Encroachment Decreases Diversity Across North American Grasslands and Savannas.” Ecology 93, no. 4: 697–703. 22690619 10.1890/11-1199.1

[ece373739-bib-0043] Ratnam, J. , W. J. Bond , R. J. Fensham , et al. 2011. “When Is a ‘Forest’ a Savanna, and Why Does It Matter?” Global Ecology and Biogeography 20, no. 5: 653–660.

[ece373739-bib-0044] Roques, K. , T. O'connor , and A. R. Watkinson . 2001. “Dynamics of Shrub Encroachment in an African Savanna: Relative Influences of Fire, Herbivory, Rainfall and Density Dependence.” Journal of Applied Ecology 38, no. 2: 268–280.

[ece373739-bib-0045] Sankaran, M. , N. P. Hanan , R. J. Scholes , et al. 2005. “Determinants of Woody Cover in African Savannas.” Nature 438, no. 7069: 846–849. 16341012 10.1038/nature04070

[ece373739-bib-0046] Scholes, R. J. , and S. R. Archer . 1997. “Tree‐Grass Interactions in Savannas.” Annual Review of Ecology and Systematics 28, no. 1: 517–544.

[ece373739-bib-0047] Siebert, F. , and N. Dreber . 2019. “Forb Ecology Research in Dry African Savannas: Knowledge, Gaps, and Future Perspectives.” Ecology and Evolution 9, no. 13: 7875–7891. 31346447 10.1002/ece3.5307 PMC6635924

[ece373739-bib-0048] Simon, M. F. , and T. Pennington . 2012. “Evidence for Adaptation to Fire Regimes in the Tropical Savannas of the Brazilian Cerrado.” International Journal of Plant Sciences 173, no. 6: 711–723.

[ece373739-bib-0049] Skhosana, F. V. , H. F. Thenga , M. J. Mateyisi , G. von Maltitz , G. F. Midgley , and N. Stevens . 2023. “Steal the Rain: Interception Loses and Rainfall Partitioning by a Broad‐Leaf and a Fine‐Leaf Woody Encroaching Species in a Southern African Semi‐Arid Savanna.” Ecology and Evolution 13, no. 3: e9868. 36937063 10.1002/ece3.9868 PMC10017313

[ece373739-bib-0050] Smit, M. , P. Malan , N. Smit , and F. Deacon . 2024. “Response of Herbaceous Vegetation in the Southern Kalahari Following a Prolonged Drought.” Journal of Arid Environments 222: 105157.

[ece373739-bib-0051] Solofondranohatra, C. L. , M. S. Vorontsova , R. A. Dewhirst , et al. 2021. “Shade Alters the Growth and Architecture of Tropical Grasses by Reducing Root Biomass.” Biotropica 53, no. 4: 1052–1062.

[ece373739-bib-0052] Staver, A. C. , S. Archibald , and S. Levin . 2011. “Tree Cover in Sub‐Saharan Africa: Rainfall and Fire Constrain Forest and Savanna as Alternative Stable States.” Ecology 92, no. 5: 1063–1072. 21661567 10.1890/10-1684.1

[ece373739-bib-0053] Staver, A. C. , C. Wigley‐Coetsee , and J. Botha . 2019. “Grazer Movements Exacerbate Grass Declines During Drought in an African Savanna.” Journal of Ecology 107, no. 3: 1482–1491.

[ece373739-bib-0054] Teague, W. , W. Trollope , and A. Aucamp . 1981. “Veld Management in the Semi‐Arid Bush‐Grass Communities of the Eastern Cape.” Proceedings of the Annual Congresses of the Grassland Society of Southern Africa 16, no. 1: 23–28.

[ece373739-bib-0055] Van Coller, H. , F. Siebert , P. Scogings , and S. Ellis . 2018. “Herbaceous Responses to Herbivory, Fire and Rainfall Variability Differ Between Grasses and Forbs.” South African Journal of Botany 119: 94–103.

[ece373739-bib-0056] Veldman, J. W. 2016. “Clarifying the Confusion: Old‐Growth Savannahs and Tropical Ecosystem Degradation.” Philosophical Transactions of the Royal Society, B: Biological Sciences 371, no. 1703: 20150306. 10.1098/rstb.2015.0306 PMC4978865 27502372

[ece373739-bib-0057] Veldman, J. W. , E. Buisson , G. Durigan , et al. 2015. “Toward an Old‐Growth Concept for Grasslands, Savannas, and Woodlands.” Frontiers in Ecology and the Environment 13, no. 3: 154–162.

[ece373739-bib-0058] Ward, J. H., Jr. 1963. “Hierarchical Grouping to Optimize an Objective Function.” Journal of the American Statistical Association 58, no. 301: 236–244.

[ece373739-bib-0059] Wasilewska‐Dębowska, W. , M. Zienkiewicz , and A. Drozak . 2022. “How Light Reactions of Photosynthesis in C4 Plants Are Optimized and Protected Under High Light Conditions.” International Journal of Molecular Sciences 23, no. 7: 3626. 35408985 10.3390/ijms23073626 PMC8998801

[ece373739-bib-0060] Wieczorkowski, J. D. , and C. E. Lehmann . 2022. “Encroachment Diminishes Herbaceous Plant Diversity in Grassy Ecosystems Worldwide.” Global Change Biology 28, no. 18: 5532–5546. 35815499 10.1111/gcb.16300 PMC9544121

[ece373739-bib-0061] Wigley, B. J. , W. J. Bond , and M. T. Hoffman . 2010. “Thicket Expansion in a South African Savanna Under Divergent Land Use: Local vs. Global Drivers?” Global Change Biology 16, no. 3: 964–976.

[ece373739-bib-0062] Wragg, P. D. , T. Mielke , and D. Tilman . 2018. “Forbs, Grasses, and Grassland Fire Behaviour.” Journal of Ecology 106, no. 5: 1983–2001.

[ece373739-bib-0063] Yarranton, G. , and R. G. Morrison . 1974. “Spatial Dynamics of A Primary Succession: Nucleation.” Journal of Ecology 62: 417–428.

